# Increased Risk for Vitamin D Deficiency in Obese Children with Both Celiac Disease and Type 1 Diabetes

**DOI:** 10.1155/2014/561351

**Published:** 2014-12-04

**Authors:** Nithya Setty-Shah, Louise Maranda, Benjamin Udoka Nwosu

**Affiliations:** ^1^Department of Pediatrics, University of Massachusetts Medical School, 55 Lake Avenue North, Worcester, MA 01655, USA; ^2^Department of Quantitative Health Sciences, University of Massachusetts Medical School, 55 Lake Avenue North, Worcester, MA 01655, USA; ^3^Division of Endocrinology, Department of Pediatrics, University of Massachusetts Medical School, 55 Lake Avenue North, Worcester, MA 01655, USA

## Abstract

*Background*. It is unknown whether the coexistence of type 1 diabetes (T1D) and celiac disease (CD) increases the risk for vitamin D deficiency.* Aims*. To determine the vitamin D status and the risk for vitamin D deficiency in prepubertal children with both T1D and CD compared to controls, TID, and CD.* Subjects and Methods*. Characteristics of 62 prepubertal children of age 2–13 y with either CD + T1D (*n* = 22, 9.9 ± 3.1 y), CD only (*n* = 18, 8.9 ± 3.3 y), or T1D only (*n* = 22, 10.1 ± 2.8 y) were compared to 49 controls of the age of 8.0 ± 2.6 years. Vitamin D deficiency was defined as 25(OH)D < 50 nmol/L, overweight as BMI of >85th but <95th percentile, and obesity as BMI > 95th percentile.* Results*. The 4 groups had no difference in 25(OH)D (ANOVA *P* = 0.123) before stratification into normal-weight versus overweight/obese subtypes. Following stratification, 25(OH)D differed significantly between the subgroups (*F*_(3,98)_ = 10.109, ANOVA *P* < 0.001). Post-hoc analysis showed a significantly lower 25(OH)D in the overweight/obese CD + T1D compared to the overweight/obese controls (*P* = 0.039) and the overweight/obese CD (*P* = 0.003). Subjects with CD + T1D were 3 times more likely to be vitamin D deficient (OR = 3.1 [0.8–11.9], *P* = 0.098), compared to controls.* Conclusions*. The coexistence of T1D and CD in overweight/obese prepubertal children may be associated with lower vitamin D concentration.

## 1. Introduction

There is considerable clinical and pathogenic overlap between type 1 diabetes (T1D) and celiac disease (CD) [1]. These two autoimmune diseases have similar genetic background and trigger mechanisms [2]. The common genetic basis for the expression of both diseases derives from the identification of human leucocyte antigen (HLA) class II molecules, DQ8 and DQ2 as key genetic risk factors for T1D and CD [3]. These diseases also share similar non-HLA risk factors, such as viral infection, effect of gut microbiome, duration of breastfeeding, and the timing of the introduction of solid foods [1].

CD is an autoimmune-mediated, chronic inflammatory disorder of the small intestine that is principally induced in genetically susceptible individuals by the ingestion of proline- and glutamine-rich proteins contained in wheat, rye, and barley, although other factors seem to play a secondary role [4]. It affects about 0.5–0.1% the population [5] but occurs in patients with T1D at a prevalence rate of 8% [6, 7].

T1D is an endocrine disorder caused by autoimmune destruction of the pancreatic beta cells leading to insulin deficiency [8]. It has a prevalence rate of 2.55 per 1000 in white youth and 0.35 per 1000 among American Indian youth in the USA [9]. An association between T1D and vitamin D physiology was suggested by Ponsonby et al. who reported that the pathogenesis of T1D may be dependent on vitamin D receptor variants [10], while studies by Bener et al. [11] and Tunc et al. [12] showed that children with T1D have lower serum 25(OH)D concentration and that these vitamin D-deficient children often require increased amounts of insulin to maintain normal glycemic control [12].

Though vitamin D deficiency has been described in patients with CD [13, 14] and T1D [15], there are no data on the synergistic impact of both CD and T1D on vitamin D status in prepubertal children. Equally, the effect of increasing adiposity on vitamin D status in patients with both CD and T1D is unclear. Recent reports indicate that 19% of children with T1D are overweight and 5.2% are obese [16]. Similarly, the prevalence of overweight and obesity at the time of diagnosis of CD is 8.8–20.8% and up to 6%, respectively [17].

Several studies have reported lower serum vitamin D concentrations in overweight/obese children compared to normal-weight children [18–21]. Our group reported a nonsignificantly lower serum 25(OH)D level in obese children with CD compared to normal-weight children with CD [22].

Despite the description of vitamin D deficiency independently in obesity, T1D, and CD, no study has explored the synergistic impact of these three pathological states on vitamin D status in prepubertal children. Such a study will provide important data to guide recommendations for adequate vitamin D supplementation in this population.

The study's primary aim was to investigate the vitamin D status and the risk for vitamin D deficiency in subjects with T1D + CD compared to controls, T1D, and CD only. We hypothesized that the coexistence of T1D and CD would be associated with significantly lower 25(OH)D concentration and increased risk of vitamin D deficiency compared to controls, T1D, and CD group.

## 2. Subjects and Methods

### 2.1. Ethics Statement

The study protocol was approved by the University of Massachusetts Institutional Review Board. All patient records and information were anonymized and deidentified prior to analysis.

### 2.2. Control Subjects

The control group (*n* = 49) was drawn from a study to evaluate the role of vitamin D status on bone mineral density. Its clinical trial identification number is NCT00756899. Written informed consent was obtained from each subject's parent or legal guardian and assent was obtained from each subject prior to participating in the study.

The control group consisted of healthy children who were recruited by means of advertisement using paper flyers distributed to the primary care physician offices in Central New England, USA. Fifty subjects signed consent for the study. Forty-nine subjects (28 males and 21 females) between 3 and 12 years of age were studied. The mean age of the cohort was 7.95 ± 2.63 years; mean age of females 7.09 ± 2.37 years; mean age of males 8.59 ± 2.68 years. All participants were of prepubertal status: males, with testicular volume of ≤3 cc, and females with Tanner stage 1 breasts as determined by palpation by the principal investigator. Subjects were excluded if they had any known metabolic or genetic diseases resulting in obesity such as severe hypothyroidism, pseudohypoparathyroidism, or Cushing's disease. We also excluded patients with systemic illnesses such as diabetes mellitus, nephrotic syndrome, chronic renal disease, or chronic inflammatory disorders. Subjects with a history of significant weight loss or gain (change of >10% body weight in 6 months) were excluded from the study. Methods used for exclusion included history, physical examination, and screening laboratory tests for fasting blood glucose, cortisol, urinalysis, comprehensive metabolic panel, serum creatinine, and thyroid function tests.

### 2.3. Study Subjects

The study group (*n* = 62) consisted of prepubertal children of ages 3–12 years who were diagnosed with either CD (*n* = 18), or T1D alone (*n* = 22), or with both diseases (CD + T1D (*n* = 22)) at the UMass Memorial Medical Center between 2008 and 2013.

Subjects were included in the study group if they had diagnoses of T1D, CD, or a combination of both diseases. Subjects that were included in the CD cohort had clinical symptoms of CD, positive serological studies, and confirmatory upper gastrointestinal biopsies consistent with the diagnosis of CD. Subjects with T1D were included in the study if they had a diagnosis of diabetes for >6 months. The diagnosis of T1D was based on fasting blood glucose of ≥126 mg/dL, and/or 2 hour postprandial glucose of ≥200 mg/dL, and/or random blood glucose of ≥200 mg/dL with symptoms of polyuria, and/or polydipsia. All subjects with T1D possessed one or more of the T1D-associated autoantibodies (insulin, islet cell, glutamic acid decarboxylase, and insulinoma associated 2). All were treated with insulin monotherapy.

All study subjects were of prepubertal status. Children in the CD + T1D and the CD-only cohort had no history of other malabsorptive disorders except CD.

A further inclusion criterion was the availability of 25(OH)D level measured six months or more after the diagnosis of CD or T1D while being on no vitamin D supplementation. The estimation of 25(OH)D at clinic visits is a routine practice at the Children's Medical Center of the UMass Memorial Medical Center. The six-month cut-off criterion for inclusion in the study ensured that all subjects were in the established phase of their clinical conditions: subjects with CD were on gluten-free diet (GFD) and patients with T1D were beyond the honeymoon phase of the clinical history of diabetes mellitus.

Subjects were excluded if they had diseases of calcium- or vitamin D metabolism, or were receiving calcium, vitamin D or multivitamin supplementation. Ten patients were excluded because they were receiving vitamin D supplements at the time of serum 25(OH)D estimation.

## 3. Study Methods

Participants in the control group were evaluated between 0800 and 1100 hours following an overnight fast, whereas the study subjects were evaluated at different times of the day.

### 3.1. Anthropometry

Height was measured to the nearest 0.1 cm using a wall-mounted stadiometer (Holtain Ltd, Crymych, Dyfed, UK) that was calibrated daily. Weight was measured to the nearest 0.1 kg using an upright scale. BMI was derived using the formula weight/height^2^ (kg/m^2^) and expressed as* z*-score for age and gender based on National Center for Health Statistics (NCHS) data [23]. Anthropometric data were expressed as mean ± SD.

### 3.2. Biochemical Study

A single venous blood sample was collected for serum 25(OH)D estimation between 0800 and 0900 hours in the control subjects, and at various times of the day for the study subjects.

### 3.3. Assay

Serum levels of 25(OH)D were analyzed using 25-hydroxy chemiluminescent immunoassay (DiaSorin Liaison; Stillwater, Minnesota), which has a 100% cross reactivity with both metabolites of 25(OH)D, namely, 25(OH)D_2_ and 25(OH)D_3_, and thus measures total serum 25(OH)D content. Its functional sensitivity is 10 nmol/L (4 ng/mL), and its intra- and interassay coefficients of variation are 5% and 8.2%, respectively. Vitamin D status was classified according to the American Academy of Pediatrics and the Institutes of Medicine criteria as deficient, 25(OH)D < 50 nmol/L (<20 ng/mL), or sufficient, 25(OH)D > 50 nmol/L (>20 ng/mL) [24]. Because vitamin D status could vary with sunlight exposure and the seasons, we categorized each subject's visit according to the seasons as follows: fall (September 22–December 21), winter (December 22–March 21), spring (March 22–June 21), and summer (June 22–September 21) [25].

### 3.4. Statistical Analyses

Statistical analyses were performed using the SPSS predictive analytics software version 22 (IBM Corporation, Armonk, NY, USA). Normal probability plots were constructed with the variables of interest. No departure from normality was detected, allowing for the use of parametric tests, without the need for transformation. Means, standard deviations, and percentages were calculated for descriptive summary statistics. Proportions were compared using Chi square tests. To adjust for the influence of adiposity on 25(OH)D concentration, subjects were stratified into normal weight versus overweight/obese groups. The rationale for this stratification is because obesity is associated with vitamin D deficiency, as vitamin D is subject to either sequestration [26] or volumetric dilution [27] in fat depots. Overweight was defined as BMI of ≥85th but <95th percentile, while obesity was defined as a BMI of > 95th percentile for age and sex. Differences between the 8 subgroups (normal weight versus overweight/obese controls, T1D, CD, T1D + CD subjects) were first explored using a two-way ANOVA, followed by post-hoc comparisons. Logistic models were fitted to the dataset to estimate adjusted odds ratios using age, BMI SDS, seasons, and race as possible confounders of the relationship between vitamin D and disease states, namely, T1D, CD, or T1D + CD.

## 4. Results

Table 1 shows the mean (±SD) values for age, gender, seasonality, anthropometric, and biochemical parameters of the study subjects and controls. The control subjects had significantly higher weight and BMI* z*-score compared to the study subjects (ANOVA *P* = 0.042), while patients with T1D were older than the other groups. There were no significant differences in height SDS, seasons, gender, and race.

**Figure 1:**
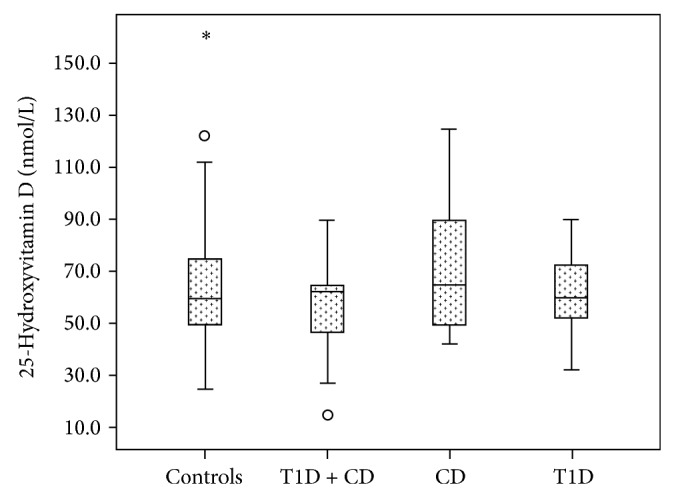
Box plots showing no significant difference in the serum concentration of 25-hydroxyvitamin D between the controls (65.3 ± 26.0 nmol/L) T1D + CD (57.5 ± 18.4), CD (74.4 ± 27.2), and T1D (61.0 ± 15.1), ANOVA *P* = 0.123, before stratification into normal-weight versus overweight/obese subgroups by body mass index criteria.

Figure 1 shows no significant difference in serum 25(OH)D concentration between the controls ((65.3 ± 26.0 nmol/L), T1D + CD (57.5 ± 18.4 nmol/L), CD (74.4 ± 27.2 nmol/L), and T1D (61.0 ± 15.1 nmol/L), (ANOVA *P* = 0.123)), before adjusting for adiposity using body mass index criteria.

**Figure 2:**
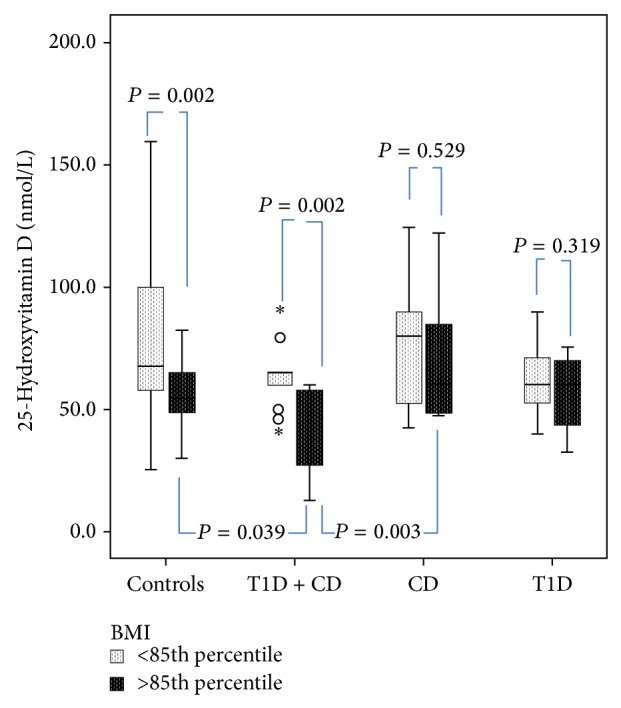
Box plots of the comparative analysis of the differences in serum 25-hydroxyvitamin D concentration of the control and study groups after stratification by body mass index criteria into normal-weight versus overweight/obese groups. The overweight/obese patients with T1D + CD (*n* = 5) had significantly lower 25(OH)D concentration compared to the overweight/obese controls (*n* = 28) (36.9 ± 20.7 nmol/L versus 55.6 ± 13.9, *P* = 0.039) and CD only (*n* = 8) (36.9 ± 20.7 versus 69.7 ± 28.6, *P* = 0.003). There was no significant difference in serum 25(OH)D concentration between the normal-weight subgroups.

In Figure 2, following adjustment for adiposity using BMI criteria to subdivide each group into normal weight and overweight/obese subgroups, there was a significant difference in serum 25(OH)D across the 8 subgroups (*F*_(3,98)_ = 10.109, *P* < 0.001). Post-hoc analysis showed significant differences in serum 25(OH)D concentration between the normal weight versus overweight/obese controls (78.1 ± 32.6 nmol/L versus 55.6 ± 13.9, *P* = 0.002) and the normal-weight versus overweight/obese patients with T1D + CD (*n* = 5) (63.6 ± 12.9 versus 36.9 ± 20.7, *P* = 0.002), but not among those with isolated CD (*n* = 8) (*P* = 0.529) or T1D (*n* = 7) (*P* = 0.319).

An analysis of the overweight/obese subjects in each group showed that the overweight/obese patients with T1D + CD (*n* = 5) had significantly lower 25(OH)D concentration compared to the overweight/obese controls (*n* = 28) (36.9 ± 20.7 nmol/L versus 55.6 ± 13.9, *P* = 0.039), CD-only cohort (*n* = 8) (36.9 ± 20.7 versus 69.7 ± 28.6, *P* = 0.003), and nearly so for T1D-only cohort (*n* = 7) (36.9 ± 20.7 versus 56.2 ± 17.4, *P* = 0.075). An analysis of the normal-weight subjects showed no significant difference in serum 25(OH)D concentration between the subgroups. Further analysis revealed a significant difference in serum 25(OH)D concentration between the normal weight versus overweight/obese in both the controls (*P* = 0.002) and the CD + T1D (*P* = 0.001), but neither in the CD nor in the TID-only group.

There was a significant inverse relationship between serum 25(OH)D concentration and BMI SDS in both the T1D + CD group (*r* ^2^ = 0.443, *β* = −0.604, *P* = 0.003) and the control group (*r* ^2^ = 0.120, *β* = −0.311, *P* = 0.027) but neither in the CD (*r* ^2^ = −0.184, *β* = −0.033, *P* = 0.905) nor in the T1D cohort (*r* ^2^ = 0.150, *β* = −0.222, *P* = 0.306).

Logistic model analysis showed that subjects with CD + T1D were three times more likely to be vitamin D deficient compared to controls (OR = 3.1 [0.8 to 11.9], *P* = 0.098), whereas subjects with isolated T1D (OR = 1.49 [0.3 to 7.8], *P* = 0.641) or CD (OR = 1.50 [0.4 to 6.0], *P* = 0.567) were 1.5 times more likely to be vitamin D deficient than controls.

## 5. Discussion

Though T1D is associated with other autoimmune diseases such as CD, Hashimoto's thyroiditis, Graves' disease, Addison's disease, and myasthenia gravis [28], CD has a malabsorptive component that could potentially result in hypovitaminosis D. The role of intestinal epithelial damage on vitamin D absorption in prepubertal children with CD has not been fully studied. Even though vitamin D receptors are present in the crypts of intestine in CD, it is unclear whether the intestinal inflammation associated with CD leads to malabsorption of fat soluble vitamins and consequent vitamin D deficiency [29]. Vitamin D deficiency has been reported in related autoimmune diseases such as T1D and chronic lymphocytic thyroiditis [30, 31], which are frequently seen in patients with CD [31]. Some investigators have speculated that the intestinal damage in CD could result in vitamin D malabsorption and vitamin D deficiency [13, 14] while others found no such evidence [22, 32].

This study, which examined the combined effects of T1D, CD, and adiposity on vitamin D status in prepubertal children, found a higher occurrence of vitamin D deficiency in children with T1D + CD (27.3%) compared to the controls (18.4%), CD (22.2%), and T1D (13.6%). It also found that the overweight/obese patients with T1D + CD had significantly lower 25(OH)D concentration compared to the overweight/obese controls, the CD only, and nearly so for T1D. In contrast, the normal-weight subjects showed no significant difference in their serum 25(OH)D concentration between the subgroups. Some of the proposed reasons for vitamin D deficiency in the obese state include sequestration of vitamin D in excess body fat, negative feedback from an elevated 1-25-dihydroxyvitamin D level, poor diet, or an avoidance of sunlight [19–21]. Vitamin D deficiency may occur in patients with CD who are placed on strict GFD as a result of variable degrees of noncompliance with the GFD recommendations. This results in the persistence of the intestinal inflammation and relative malabsorption of fat soluble vitamins, including vitamin D. Low serum vitamin D concentration can also result from the sequestration of vitamin D in fat depots in patients with CD who regained or exceeded their body weight as a result of strict compliance with GFD.

Finally, the logistical model analysis showed that subjects with CD + T1D were three times more likely to be vitamin D deficient compared to controls, whereas subjects with either T1D or CD alone were only 1.5 times more likely to be vitamin D deficient than controls.

Taken together, it is likely that both the severity and the prevalence of vitamin D deficiency will increase with time in patients with both T1D and CD given the high prevalence (67%) of malnutrition in patients with CD alone [13], along with the increasing prevalence of overweight (19%) and obesity (5%) [33] in this population. In concert with published reports, vitamin D deficiency was present in both the overweight/obese subjects and overweight/obese controls. However, the significantly reduced serum 25(OH)D concentration in the overweight/obese subjects with CD + T1D compared to the overweight/obese controls (*P* = 0.039) is suggestive of an additional vitamin D attenuating effect from the coexistence of CD and T1D in an individual. Proposed mechanisms include malabsorption from intestinal inflammation in patients with CD who are not fully compliant with GFD, and/or the effect of narrow dietary choices, and dietary restrictions in patients with CD + T1D, which could limit optimal vitamin D intake.

Additionally, though gluten-free foods have similar glycemic indices as normal foods [34], they may be less favorable in patients with T1D who need to balance caloric intake [35]. This is because patients on GFD appear to have a lesser degree of satiety and in turn tend to increase the amount of gluten-free foods consumed [35]. Because gluten-free foods are rich in fats, the resultant increases in weight gain and adiposity may lead to the sequestration of vitamin D in fat depots and consequent vitamin D deficiency. It is therefore necessary to ensure normal vitamin D status in these patients by increased surveillance for hypovitaminosis D.

This study has some limitations. First, the cross-sectional study design limits causal inference on the effects of seasons, race, and biochemical parameters on vitamin D status. Second, we did not administer a food recall to accurately determine vitamin D content of the subjects' diet. This is important as it could be argued that the diets in overweight/obese persons with CD + T1D could be the major reason for the lower vitamin D levels in these subjects. However, if reduced vitamin D intake was the primary reason for the low vitamin D levels in these patients, then the obese/overweight subjects with either CD or T1D only would equally have significantly lower 25(OH)D concentration. Third, we did not evaluate other components of the complex vitamin D metabolic pathway, such as parathyroid hormone (PTH). This is relevant because PTH could be elevated in states of vitamin D deficiency and hypocalcemia. However, 25(OH)D is the major circulation form of vitamin D and its stability in plasma and long half-life (>15 days) make it a highly sensitive and specific marker of vitamin D status [36]. Fourth, our results were derived from a tertiary care center, located at latitude 42°N in a single state in the United States of America; therefore, we are uncertain that our results are generalizable to other centers, states, or countries.

One of the strengths of this study is that it was conducted exclusively in prepubertal children. Studies in prepubertal children are a better guide to potential causal associations than studies in pubertal or postpubertal subjects. This is because associations in childhood are less prone to confounding physiologic and lifestyle factors, such as the different stages of pubertal maturation, and the effects of fluctuations in pubertal hormone levels on growth and adiposity. This prepubertal cohort represents the youngest group of subjects in whom the risk for vitamin D deficiency in patients with T1D + CD could be demonstrated.

Additionally, the robust group of healthy prepubertal children in the control group ensured the validity of the anthropometric and biochemical comparisons. This study's case-controlled design enabled us to evaluate a sizeable cohort of patients with T1D, CD, or both and compare their results to a control group. This sample size enhanced the detection of subtle differences between the groups of interest. This sample also contained a fair representation of the fractional composition of each of the major racial groups in Central Massachusetts, thus enabling us to analyze the effects of differential insolation on racial groups. The fact that this study was conducted exclusively amongst subjects living at the same geographical latitude (42°N) ensured uniformity of exposure to solar radiation. Phlebotomy was performed at different seasons of the year, thus ensuring that seasonality was not a confounder. All anthropometric data were expressed as* z*-score, all analyses were adjusted for covariates, and skewed data were log transformed before analysis.

## 6. Conclusions

The coexistence of T1D and CD in overweight/obese prepubertal children may be associated with lower serum vitamin D concentration compared to healthy controls. Further research is needed to confirm our findings.

## Figures and Tables

**Table 1:** A comparative analysis of the characteristics of the subjects and controls.

Parameters	Controls	CD + T1D	CD only	T1D only	*P* value
Number of subjects	49	22	18	22	
Age (years)	7.95 ± 2.63	9.92 ± 3.09	8.92 ± 3.32	10.13 ± 2.78	0.009
Sex: males (%)	57.1 (28/49)	27.3 (6/22)	44.4% (8/18)	54.5% (12/22)	0.118^*^
Weight SDS	1.05 ± 1.97	0.25 ± 0.96	0.06 ± 1.57	0.62 ± 0.68	0.067
Height SDS	0.19 ± 1.72	−0.03 ± 1.02	−0.70 ± 1.44	0.35 ± 0.92	0.095
BMI SDS	1.26 ± 1.62	0.41 ± 0.94	0.48 ± 1.55	0.63 ± 0.93	0.042
25(OH)D nmol/L	65.25 ± 26.03	57.5 ± 18.41	74.42 ± 27.22	61.01 ± 15.07	0.123
25(OH)D <50 nmol/L	9/49 (18.4%)	6/22 (27.3%)	4/18 (22.2%)	3/22 (13.6%)	0.699
BMI > 85th percentile (%)	57.1% (28/49)	22.7% (5/22)	44.4% (8/18)	32.7% (7/22)	0.032^*^
Race: white including Hispanics (%)	89.8% (44/49)	95.5% (21/22)	88.9% (16/18)	95.5% (21/22)	0.740^*^
Season (winter + spring)	65.3% (32/49)	54.5% (12/22)	50% (9/18)	40.9% (9/22)	0.256^*^

CD: celiac disease; T1D: type 1 diabetes; 25(OH)D: 25-hydroxyvitamin D; BMI: body mass index; SDS: standard deviation score; ^*^ *P* value calculated using Chi square for proportions; and ANOVA for means of continuous values. All values expressed as mean ± standard deviation.

## Abbreviations

CD: Celiac disease
T1D: Type 1 diabetes
25(OH)D: 25-Hydroxyvitamin D
SD: Standard deviation
ANOVA: Analysis of variance
PTH: Parathyroid hormone.
